# Is automatic detection of hidden knowledge an anomaly?

**DOI:** 10.1186/s12859-019-2815-4

**Published:** 2019-05-29

**Authors:** Judita Preiss

**Affiliations:** University of Salford, The School of Computing, Science & Engineering, Newton Building, Salford, Greater Manchester, M5 4WT UK

**Keywords:** Literature based discovery, Anomaly detection, Unified medical language system

## Abstract

**Background:**

The quantity of documents being published requires researchers to specialize to a narrower field, meaning that inferable connections between publications (particularly from different domains) can be missed. This has given rise to automatic literature based discovery (LBD). However, unless heavily filtered, LBD generates more potential new knowledge than can be manually verified and another form of selection is required before the results can be passed onto a user. Since a large proportion of the automatically generated hidden knowledge is valid but generally known, we investigate the hypothesis that non trivial, interesting, hidden knowledge can be treated as an anomaly and identified using anomaly detection approaches.

**Results:**

Two experiments are conducted: (1) to avoid errors arising from incorrect extraction of relations, the hypothesis is validated using manually annotated relations appearing in a thesaurus, and (2) automatically extracted relations are used to investigate the hypothesis on publication abstracts. These allow an investigation of a potential upper bound and the detection of limitations yielded by automatic relation extraction.

**Conclusion:**

We apply one-class SVM and isolation forest anomaly detection algorithms to a set of hidden connections to rank connections by identifying outlying (interesting) ones and show that the approach increases the *F*_1_ measure by a factor of 10 while greatly reducing the quantity of hidden knowledge to manually verify. We also demonstrate the statistical significance of this result.

## Background

Literature based discovery (LBD) attempts to automatically address the fact that the volume of publications produced daily forces researchers to restrict the number of articles they read, potentially resulting in inferable connections being missed – for example, in the biomedical domain, Swanson [1] found one publication mentioning *Raynaud disease* as affecting *blood viscosity*, *platelet aggregation*, and *vascular reactivity*, and another stating that *fish oil* has the opposite effect on the same, but the connection between *Raynaud disease* and *fish oil* had not been noticed. This forms the outline of the *A-B-C* model [1] which extracts all pairs of *A* and *B* that are known to be related (such as *Raynaud disease* - *blood viscosity*) and matches over *B* terms to find connections *A* - *B* - *C* where *A* - *B* appear in one publication and *B* - *C* in another but no single publication connects *A* directly to *C*.

However, this model proposes a high proportion of everyday knowledge of the domain [2] as well as a high number of spurious connections: for example, publications describing clinical trials will frequently mention *patients*, *trials* or *weeks*, but connecting through any such *B* terms will lead to a very large number of (meaningless) connections. To avoid this problem, systems often carry out heavy filtering: some options include restricting the time period from which the data is drawn (e.g. [3]), manually or semi-automatically creating stoplists (e.g. [4]), restricting the types of terms or relations extracted (e.g. [5]), or only using publications’ titles (e.g. [1]). All such restrictions can lead to important inferable connections being missed.

A possible addition, or alternative, to filtering is re-ranking of the resulting proposed connections: instead of returning all the proposed hidden knowledge to a user unordered, the connections are ordered by likelihood of being an ‘interesting’ (non trivial, e.g. requiring clinicial trials to ensure validity) hidden knowledge pair. Amongst others, the order can be determined by the number of linking (*B*) terms (LTs, e.g. [6]), computed confidence values (e.g. [7]), or by assigning weights and rankings to the LTs based on medical subject headings (e.g. [4]).

We propose using anomaly detection to annotate potentially interesting connections: i.e. we hypothesize that these connections can be identified as outliers among a vast quantity of correct, but uninteresting, connections. To our knowledge, this is the first application of anomaly detection to LBD. Moreover, an isolation forest implementation of anomaly detection [8] has linear time complexity with a low memory requirement, allowing an LBD system to be employed with minimal filtering reducing the number of incorrectly discarded knowledge pairs.

### Literature based discovery

Swanson’s [1] *A-B-C* approach outlined above has remained a central method for LBD. This work employs this model and focuses on **open discovery**, where all *B* terms connected to the term of interest *A* are pursued to find a reachable set of concepts *C*, rather than **closed discovery** where a connection is already suspected between given terms *A* and *C* and only the linking terms, the *B* terms, are sought.

The approach relies on the relations used – if the connection between *A* and *B* is incorrect, or not significant for single step LBD purposes (e.g. HAS PRECISE INGREDIENT), the inferred connections will either not be meaningful (in the first case) or novel (in the second case). Automatically extracted relations lead to a large variation in the quality and quantity of hidden knowledge generated depending on the type of relation used – for example, Preiss et al. [9] show that refining the relation (for example basing them on linguistic principles rather than simple co-occurrence) significantly reduces the quantity of spurious relations produced. We propose two evaluations: (1) using the relations contained in the Unified Medical Language System (UMLS) metathesaurus (which are manually identified), and (2) the employment of the SemRep system [10] which automatically extracts subject-relation-object triples (such as *X treats Y*) from biomedical text using underspecified syntactic processing and UMLS domain knowledge.

The UMLS metathesaurus contains inter-concept relationships, both hierarchical (such as ISA or PART OF), and associative (such as MAY TREAT or MAY DIAGNOSE). The hierarchical relationships are not useful for interesting single step LBD – for example if the UMLS contains *fish oil*MAY TREAT*Raynaud’s disease*, proposing the valid missing relation *fish oil*MAY TREAT*Raynaud’s phenomenon* (arising from *Raynaud’s disease*ISA*Raynaud’s phenomenon*) is not interesting. For both SemRep relations and UMLS relations, concepts related via ISA are merged and other UMLS hierarchical or part of relations are not used as features. To remove (often disused) infrequent relations a minimum number of occurrences of each relation is also imposed (for example, a minimum frequency of 10 reduces the number of 2010AB UMLS relations to 35). SemRep relations are very similar to UMLS relations, producing triples such as *cui*_*A*_MAY TREAT *cui*_*B*_. For the purposes of the A-B-C model, the relation itself is unimportant for the purposes of the A-B-C model as it is disregarded at the LBD stage. The anomaly model uses the most common relations for the input given and thus is trained separately for each version of UMLS and for each version of SemRep.

### Filtering knowledge

The hidden knowledge proposed by an LBD system forms basis for further investigation and clinical trials. It is therefore important that the most promising pieces of hidden knowledge can be identified in a manner that does not discard other, potentially useful, knowledge.

The following filtering options are employed: (1) the automatic creation of stoplists from common linking terms [11], (2) the removal of terms with a high outdegree, and (3) the restriction of relations to those useful for LBD. The first two filtering options remove terms such as *clinical trial*, while the third option removes relations from UMLS that are not useful for single step LBD (for example, *A*HAS PRECISE INGREDIENT*B* and *A*TREATS*C* will give a potentially new connection between *A* and *C*, but this is not an interesting connection) and negative relations (such as, NEG TREATS i.e. does not treat) from SemRep.

### Re-ranking and anomaly detection

To reduce the quantity of hidden knowledge pairs returned to a user (e.g. UMLS 2014AB generates 5,748,834 pairs), an order can be imposed on the hidden knowledge generated: this is often based on traditional ranking approaches such as information measure, shared connections or semantic-knowledge based ranking [12]. As an alternative, we suggest re-ranking based on an anomaly detection algorithm, as this approach is highly suitable for datasets with very small numbers of outliers (which for LBD translate to interesting pieces of hidden knowledge). It is frequently used in security, for example in fraud detection, and it has been employed within natural language processing, for example for the detection of anomalous text [13] which has a similar premise to hidden knowledge generated by an LBD system.

A number of approaches to anomaly detection exist, starting from manually created rules which are constructed by experts and are therefore difficult to maintain, to machine learning techniques which can capture correlations between features and make predictions without needing labelled data, merely based on the fact that outliers are rare. The quantity of data generated by an unfiltered LBD system may dictate the chosen anomaly detection algorithm as identification of anomalies frequently takes place in RAM.

One-class support vector machine (SVM) [14] is a novelty detection algorithm suitable for highly unbalanced datasets. It extends the original SVM methodology so that only the larger class (in this case the ‘uninteresting’ knowledge) is used for training, and new data is classified as either similar or different to the training set. To avoid potential one-class SVM memory issues, isolation forests [8] which have been shown to be similarly useful for anomaly detection while maintaining a small memory footprint are also explored. They exploit the fact that attribute-values should be very different for (the numerically small class of) anomalies, and thus when a decision tree is built these attribute-values should appear close to the root of the tree. The approach partitions the data into smaller sections, builds decision trees for these and uses path lengths within these to identify outliers.

Aside from differing memory requirements, the two approaches frame the problem differently: unlike isolation forests, one-class SVM is a novelty detection algorithm – new observations are classified as being within the regular set or not. Overall, outlier detection algorithms do not assume the existence of a clean dataset for regular data which fits better with the LBD premise than a typical classifier.

## Experiment set-up

Machine learning algorithms, including anomaly detection, use features to represent data in vector form and then create models from these representations. Terms themselves can be valuable features: for example, before the link was verified, *Raynaud disease* source term, *fish oil* target term, and *blood viscosity* linking term should have been identified as an interesting hidden connection based on the terms alone. However, two difficulties present themselves when terms are used directly: (i) a large number of terms would result in very long feature vectors, for example UMLS 2017AB contains 3,640,131 distinct terms, and training a machine learning algorithm with such input without over-training would require **very** large training corpora, (ii) not all terms are equal, for example a linking term such as *blood viscosity* is more valuable (for the identification of interesting knowledge) than *patient*.

The first problem is addressed by observing that each concept in UMLS is also assigned a broad semantic type, such as *Disease or Symptom* or *Clinical Drug*. Using these semantic types instead of terms directly results in e.g. *Raynaud disease – blood viscosity – fish oil* connection turning into *Disease or Symptom* as source term, *Pharmacologic Substance* as target term, with *Physiologic Function* as linking term (note that other broad categories, such as word embeddings, could be employed).

The solution to the second issue uses the fact that terms can be weighted differently based on their importance which can be propagated to their semantic types, and so the feature vectors. Such a weighting can be provided by, e.g., the PageRank algorithm [15] which assigns a value to each vertex in a graph depending on the probability of a random walk ending up there in a sufficiently large time. Since UMLS concepts can be viewed as the vertices of a graph, with the semantic network relationships as the edges, the PageRank algorithm can be applied to all the vertices to produce a numerical weight for each vertex (and thus term).

For each proposed hidden knowledge pair *A*−*C*, there is at least one linking term *B*_1_ such that the connections *A*−*B*_1_ and *B*_1_−*C* are known. However, there can be more than one linking term – we include the number of linking terms as a features as we hypothesize that it will be inversely correlated with interestingness value.

To summarize, for a given candidate hidden knowledge pair, *A* and *C*, with linking terms *B*_1_,…,*B*_*n*_, the chosen features are:

1. *n*, the number of linking terms.
2. *A*’s semantic type distribution (using *A*’s PageRank).
3. *C*’s semantic type distribution (again using *C*’s PageRank).
4. The distribution across semantic types of the PageRanks of all LTs.
5. A distribution over the chosen connecting relations between *A* and *B*_*i*_ and *B*_*i*_ and *C* (a sum of the *B*_*i*_’s PageRanks).

**Fig. 1:**
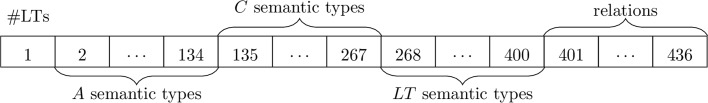
Feature array for one hidden knowledge pair (metathersaurus relations from 2010AB)

A visual representation of the features used can be seen in Fig. 1. The feature vectors are sparse, particularly the *A* and *C* sections: for example, all suggested connections from *Acetaminophen 2.71 MG/ML* will only contain its PageRank in the semantic type field corresponding to *Clinical Drug* in the first 2–134 segment of the feature array. However, separating the information regarding source and target terms allows the system to learn about useful combinations of these (such as *A*∈{*disease or symptom*} and *C*∈{*clinical drug*}).

## Results and discussion

Since the knowledge generated by an LBD system is new, there is no gold standard for evaluation. A widely accepted method for evaluation of large scale systems is timeslicing [16], which consists of selecting a date, generating hidden knowledge from data prior to this date while creating a gold standard from data after the cutoff date and comparing the generated hidden knowledge to the automatically created gold standard. Three separate cutoff dates are required for these experiments: the anomaly detection model is built from hidden knowledge generated from information up to *date*_1_ with gold standard annotation (outliers) annotated from information up to *date*_2_. The trained model is then used to classify hidden knowledge generated from information up to *date*_2_, and an evaluation is performed against information up to *date*_3_. Note that even though information up to *date*_2_ is used to classify the data for the model, there is no overlap of the anomaly detection model thus trained and the hidden knowledge generated from *date*_2_. The UMLS results are presented in Table 1 and include the size of the gold standard (|*GS*|), the original quantity of hidden knowledge proposed (orig |*HK*|) and the original *F*-measure (orig F). For UMLS, the gold standard contains pairs appearing in UMLS *date*_3_ that did not appear in UMLS *date*_2_, while for SemRep the gold standard corresponds to relations extracted from PubMed abstracts between *date*_2_ and *date*_3_ that did not appear in PubMed before *date*_2_. The pairs of results, the quantity of hidden knowledge for the isoforest (iso |*HK*|) and one-class SVM (one |*HK*|) and their F-measures are also included. The results correspond to removal of the following terms: (1) those with an outdegree exceeding 5000, or (2) occurring more than 10,000 times as linking terms. Experiments with varying outdegree values and common linking term frequency did not yield any significant differences in performance and the chosen values were selected to ensure a reasonable model training time. However, the performance (F-measure) improvement with anomaly detection was significant for both isoforest and one-class models (*p*=0.018 for isoforest and *p*=0.0015 for one-class using a paired *t*-test), and the one-class model performed significantly (*p*=0.0094) better than the isoforest model. Combined with the reduction in quantity of hidden knowledge (which is frequently around factor of 5 for the isolation forest model), these results show that anomaly detection yields significant improvement over a straight forward LBD model.

**Table 1:** UMLS results showing F-measures and quantities of **H**idden **K**nowledge from **orig**inal, **iso**lation forest and **one**class SVM generation

Train - Test - Eval	|*GS*|	Orig |*HK*|	Orig F	Iso |*HK*|	Iso F	One |*HK*|	One F
2006 - 2010 - 2013	10,237	2,104,116	0.0049	352,518	0.0055	1,986,652	0.0099
2007 - 2011 - 2014	8,851	1,914,307	0.0046	399,630	0.0068	1,800,667	0.0093
2008 - 2012 - 2015	5,476	2,094,190	0.0026	2,943	0.0045	1,964,551	0.0050
2009 - 2013 - 2016	9,040	3,547,949	0.0025	746,843	0.0030	3,407,363	0.0051
2010 - 2014 - 2017	24,772	5,748,834	0.0043	2,408,314	0.0048	5,434,823	0.0074

### Discussion

While the *F* measure based on the anomaly detection algorithm shows an improvement, it may still seem low. However, this is not an unexpected value: e.g. Preiss and Stevenson [17] obtain an F-measure between 1×10^−03^ and 3×10^−03^ for their large scale literature discovery. Analysing the precision (and thus *F* measure), the options for annotated outliers which do not appear in the gold standard are:

1. The hidden knowledge suggested should appear in UMLS but is missing.
2. The hidden knowledge generated has not yet been discovered.
3. The hidden knowledge produced is incorrect.

Note that since the hidden knowledge is generated from manually annotated UMLS relations, point 1 is ruled out.

Conversely, it is necessary to investigate pairs in the gold standard which are annotated as normal (i.e. non interesting) by the anomaly detection algorithms. A large proportion of gold standard outliers classified as normals corresponds to relationships between two concepts of the same (or closely related) semantic type, such as:

- A: *Miconazole nitrate 2% cream, top* (clinical drug)
  C: *Miconazole product* (organic chemical and pharmacologic substance)

Examples such as these are not interesting hidden knowledge, however their appearance in the training data will have an effect on the created models, and although this example was not classified as anomalous, other examples may be (and those may be missing from UMLS). The obvious refinement, removing pairs with identical semantic types, would unfortunately also remove potentially useful pairs, such as:

- A: *liver; inflammation* (disease or syndrome)
  C: *chronic active hepatitis* (disease or syndrome)

To avoid producing hidden knowledge between identical semantic types, Yetisgen-Yildiz and Pratt [5] suggest restricting an LBD system to connections between *disease* source terms and *chemicals & drugs, genes & molecular sequence* target terms, hypothesizing that it is more likely that interesting connections will appear between concepts of specific semantic types. However, restricting the semantic types reduces the gold standard (and thus also the training data) to unusable levels – the size of the gold standard before and after (Y-P) restriction are shown in Table 2.

**Table 2:** The reduction in the UMLS gold standard when Yetisgen-Yildiz and Pratt semantic type filtering is used

Train - Test - Eval	Orig |*GS*|	Y-P |*GS*|
2006 - 2010 - 2013	10,237	275
2007 - 2011 - 2014	8,851	486
2008 - 2012 - 2015	5,476	739
2009 - 2013 - 2016	9,040	235
2010 - 2014 - 2017	24,772	649

As part of the model, isolation forest produces (100) decision trees where leaf nodes appearing close to the root of the tree represent outliers, while deep tree structures show non outlier data. The trees also allow the decision points to be examined: the most common decision points are the expected *A:disease or syndrome, C:disease or syndrome, LT:disease or syndrome, number of linking terms, A:clinical drug, relation:associated_with, LT:finding, relation:may_treat*, and *C:finding*.

At a first glance, an unexpected result is the much higher performance of one-class SVM over isolation forest: one-class SVM is a novelty detection algorithm and thus seems less suitable to the problem of identification of interesting hidden knowledge than isolation forests. However, the two are suited to different types of distributions (one-class SVM being better with problems which are strongly non-Gaussian), and have different parameter sensitivities. While intuitively the data should be separable, and thus an increase in performance is expected using anomaly detection, the small quantity of training data containing the most useful patterns is most likely to blame for the small increase in performance – the hypothesis is validated, but much greater improvements are likely to be seen with the technique if better training data is supplied to the algorithm.

Similar results are obtained with automatic relations from publications using SemRep. The most recent release of the Semantic Medline database [18] (version 31_R, to 31/12/2017) was used in a 2010 - 2014 - 2017, train - test - eval, split and an isoforest F-measure improvement of 0.0024 over an original F-measure of 0.0014 was observed (unfortunately the one-class SVM model exceeds 125GB RAM and thus failed to train.). Again, the gold standard (and therefore the training data) is rather small at 5094 pairs of hidden knowledge and this is likely the cause of the low F-measure. However, the anomaly detection model is shown to also increase performance when an automatic technique for relation extraction is used.

## Conclusions and future work

Literature based discovery, an automatic method to generate inferable connections from relations, suffers from generating too many hidden connections when performed at scale. We apply one-class SVM and isolation forest anomaly detection algorithms to a set of hidden connections to rank connections by identifying outlying (interesting) ones and show that the approach significantly increases performance (*F* measure) while reducing the quantity of data passed on for manual verification. The performance is explored using manually annotated relations contained in the UMLS, but similar results are also shown to hold when an automatic relation extraction method is employed.

We hypothesise that the performance could be increased given a greater number of ‘interesting’ connections in training data, and future work includes optimization of the training, testing and evaluation splits.

## Abbreviations

LBD: Literature based discovery
LT: Linking term
SVM: Support vector machine
UMLS: Unified medical language system
